# Characterization of Platelet Function-Related Gene Predicting Survival and Immunotherapy Efficacy in Gastric Cancer

**DOI:** 10.3389/fgene.2022.938796

**Published:** 2022-06-28

**Authors:** Yan Xia, Xin Lin, Yangyang Cheng, Huimin Xu, Jingya Zeng, Wanlin Xie, Mingzhu Wang, Yihua Sun

**Affiliations:** ^1^ Department of Clinical Laboratory, Harbin Medical University Cancer Hospital, Harbin, China; ^2^ Scientific Research Center, The Second Affiliated Hospital of Harbin Medical University, Harbin, China

**Keywords:** platelet function-related gene, gastric cancer, bioinformatics analysis, prognosis, tumor microenvironment

## Abstract

Immunotherapy is widely used to treat various cancers, but patients with gastric cancer (GC), which has a high mortality rate, benefit relatively less from this therapy. Platelets are closely related to GC progression and metastasis. This study aimed to find novel potential biomarkers related to platelet function to predict GC and immunotherapy efficacy. First, based on platelet activation, signaling, and aggregation (abbreviation: function)-related genes (PFRGs), we used the least absolute shrinkage and selection operator (Lasso) regression method to construct a platelet-function-related genes prognostic score (PFRGPS). PRFGPS was verified in three independent external datasets (GSE26901, GSE15459, and GSE84437) for its robustness and strong prediction performance. Our results demonstrate that PRFGPS is an independent prognostic indicator for predicting overall survival in patients with GC. In addition, prognosis, potential pathogenesis mechanisms, and the response to immunotherapy were defined via gene set enrichment analysis, tumor mutational burden, tumor microenvironment, tumor immune dysfunction and exclusion (TIDE), microsatellite instability, and immune checkpoint inhibitors. We found that the high-PRFGPS subgroup had a cancer-friendly immune microenvironment, a high TIDE score, a low tumor mutational burden, and relatively low microsatellite instability. In the immunophenoscore model, the therapeutic effect on anti-PD-1 and anti-CTLA-4 in the high-PRFGPS subgroup was relatively low. In conclusion, PRFGPS could be used as a reference index for GC prognosis to develop more successful immunotherapy strategies.

## Introduction

Gastric cancer (GC) is a common malignancy of the digestive system with high morbidity and mortality (Sung et al., 2021). The overall survival rate of patients with GC after conventional chemotherapy is still low, especially since median survival for advanced GC is less than 1 year (Smyth et al., 2020). In recent years, the rapid rise of immunotherapy has opened new treatment prospects for patients with GC (Chivu-Economescu et al., 2018). Immunotherapy is characterized as the stimulation of specific immune responses that inhibit and kill tumor cells, thus reducing the rates of tumor recurrence and metastasis. As a new cancer treatment strategy, immunotherapy significantly improves overall survival (OS) in patients with advanced GC (Fuchs et al., 2018; Chen et al., 2020). Immune checkpoint inhibitors (ICIs) are presently the most commonly employed immunotherapy agents for cancer treatment (Galon and Bruni, 2019). However, for most cancers, only a third of patients respond to ICI treatment (Smyth et al., 2020). GC has a high degree of intratumoral heterogeneity (ITH), which is the primary cause of tumor cell resistance and survival and is thus a major obstacle to improving patient prognosis. Through multi-omics analysis, The Cancer Genome Atlas (TCGA) classified primary GC into four molecular subtypes, among which EBV-positive GC and micro satellite-instable GC have better prognosis (Cancer Genome Atlas Research Network, 2014). However, these two types are very rare in advanced GC. Patients with same tumor-node-metastasis (TNM) stratification sometimes have different prognoses; hence, patient outcomes are influenced by the chosen treatment strategy. Therefore, it is important to identify biomarkers for predicting GC and the immunotherapy outcomes.

Platelets play important roles in hemostasis and thrombosis. Platelets are considered to be “accomplices” in malignancy, as they protect circulating tumor cells from shear forces and cloak them from leukocytes by forming a thrombus around them (Mendoza-Almanza et al., 2020). Platelet-tumor-cell interactions have been identified as important factors in cancer development, progression, and metastasis (Roweth and Battinelli, 2021). Tumor cells induce platelet activation and aggregation, thereby causing thrombosis (Palacios-Acedo et al., 2019). Tumor cells also recruit platelets into the tumor microenvironment (TME), and platelets are activated by tumor cells to release the cytokines VEGF, CCL5, PDGF, TGFβ, PG, TPM3, LPA, PF4, PAF, and HGF, which promote the epithelial-mesenchymal transition of tumor cells (Mendoza-Almanza et al., 2020). VEGF and TGFβ have strong mitogenic activity, and they directly promote tumor cell growth and proliferation and enhance neovascularization, thus contributing to tumor progression and metastasis (Wojtukiewicz et al., 2017). For these reasons, platelets have now become a target for cancer therapy.

It is possible that tumor-cell-induced platelets are involved in several mechanisms of antitumor immunity, promoting an immunosuppressive TME state (Gockel et al., 2022). The platelets activated by tumor cells can directly release TGF-β and downregulate natural killer (NK) cell NKG2D receptors (Kopp et al., 2009). They can also inhibit NKG2D, NKp30, and DNAM-1 receptors in a TGFβ1-dependent manner by releasing exosomes, thereby leading to NK cell dysfunction (Sadallah et al., 2016). Tumor-cell-activated platelets modulate the immune activities of CD4^+^T, CD8^+^T, and NK cells and transform them into an immunosuppressive phenotype (Gockel et al., 2022). It has been shown that regulatory T (Treg) cells must come into contact with platelets in order to secrete the effector IL-10 (Rossaint et al., 2021). Therefore, activated platelets have been implicated as a main reason for failure of ICI treatment (Metelli et al., 2020).

Few studies have investigated the mechanisms of platelet activation, characteristics of the regulatory immune microenvironment, and the impact on immunotherapy outcome in patients with GC. This study aimed to construct a platelet-function-related genes prognostic score (PFRGPS) consisting of five platelet-function-related genes (PFRGs), using bioinformatics, and explore the relationship between the PFRGPS and TME. Furthermore, we sought to examine the relationship between PFRG expression profiles and immunotherapy. Our findings can provide an effective strategy for improving the stratification of patients with GC, ultimately promoting the development of personalized treatments.

## Materials and Methods

### Data Collection and Preprocessing

**FIGURE 1:**
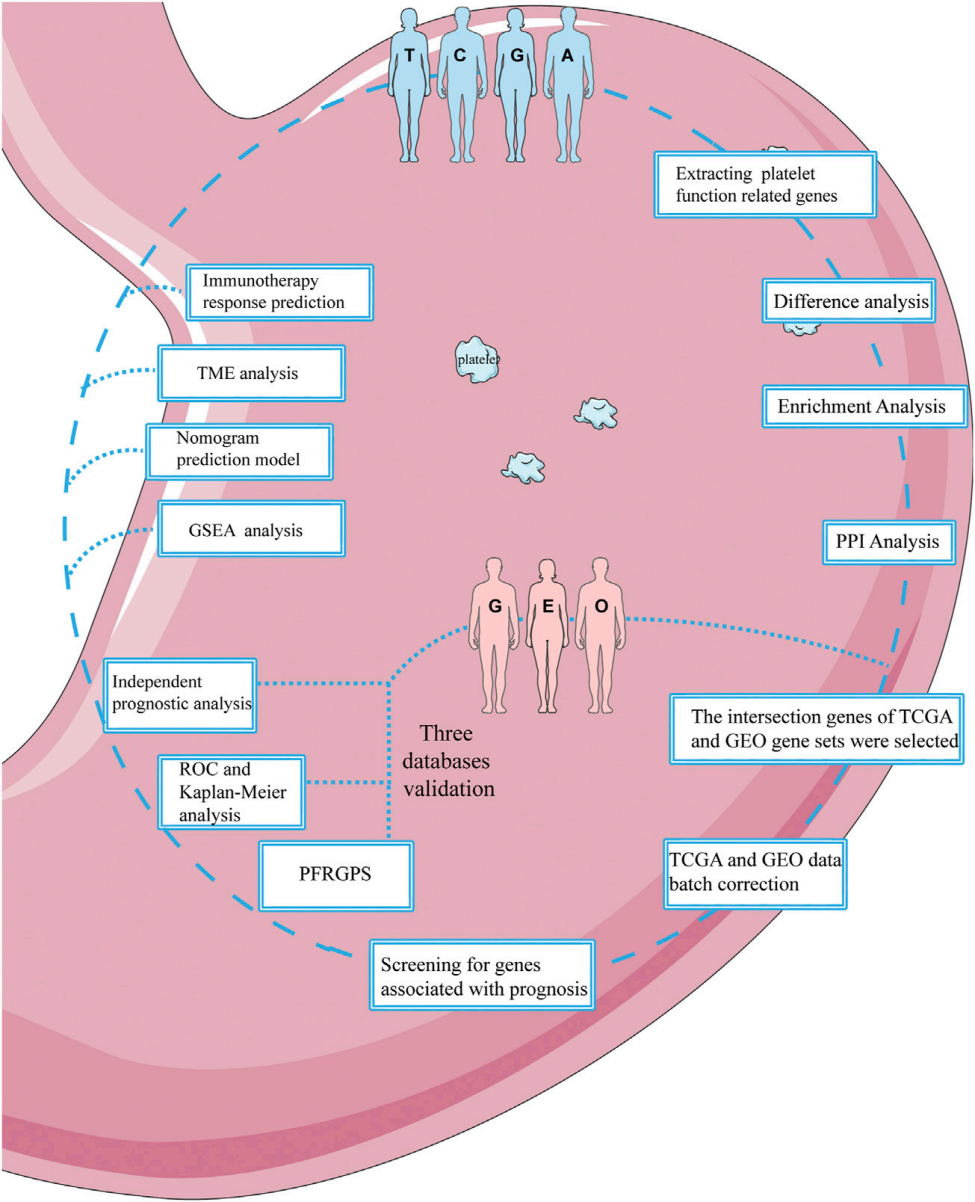
Flowchart of this study. This figure was produced with the assistance of Servier Medical Art (https://smart.servier.com).

The flow chart is shown in Figure 1. Platelet activation, signaling and aggregation (abbreviation: function), related genes (*n* = 261) were downloaded from the Gene Set Enrichment Analysis (GSEA) (http://www.gsea-msigdb.org/gsea/index.jsp). RNA-Seq data and complete clinical, survival, and somatic mutation information of patients with GC (375 tumor samples and 32 normal samples) were obtained from TCGA (https://portal.gdc.cancer.gov/). In addition, external validation cohorts GSE26901 (*n* = 109), GSE15459 (*n* = 192), and GSE84437 (*n* = 433) are from the Gene Expression Omnibus (GEO) (https://www.ncbi.nlm.nih.gov/geo/). To ensure the accuracy of the study, patients with 0 days of follow-up were excluded before the establishment of the prognostic model. The clinical features of patients who met the requirements of the model are listed in Supplementary Table S1.

### Construction of Platelet-Function-Related Genes Prognostic Score and Calculation of the Prognostic Score

The filter |log2FC| > 1 was used for fold change (FC), and the false discovery rate (FDR) was set as < 0.05. Next, 261 PFRGs were crossed with differential GC genes, and 38 differentially expressed platelet-function-related genes (DEPFRGs) were obtained. The analysis used the Gene Ontology (GO) and Kyoto Encyclopedia of Genes and Genomes (KEGG) pathways with the R packages “clusterProfiler,” “org.Hs.eg.db,” and “enrichplot.” Visualization of enrichment analysis was realized with the R packages “ggplot2” and “GOplot.” Protein-protein interaction (PPI) network construction was carried out with String (https://www.string-db.org/).

Univariable Cox regression analysis was used to determine prognosis-related PFRGs (PPFRGs), and then the R package “ConsensusClusterPlus” was used to draw a waterfall plot to show PPFRG mutations. A Circos plot was drawn using the R package “Rcircos” to show similarities and differences in the microscopic characteristics of PPFRGs. Least absolute shrinkage and selection operator (Lasso) regression analyses were used to determine potential predictors of non-zero coefficients so as to select the best OS-related PPFRGs (Tibshirani, 1997). Next, a 10-fold cross-validation was performed to determine the core genes ultimately used to construct the PFRGPS system (Friedman et al., 2010). We constructed a prognostic risk score formula based on the weighted linear combination of corresponding mRNA expression levels and corresponding regression coefficients obtained *via* Lasso regression analysis. The formula used to calculate PFRGPS was as follows:

$$\mathrm{PFRGPS=}\sum_{\mathrm{i=1}}^{n}\mathrm{coef}\left(\mathrm{genei}\right)*\mathrm{expr}\left(\mathrm{genei}\right)$$

### Validation of Platelet-Function-Related Genes Prognostic Score

We used data from TCGA database as a training cohort. We divided 350 patients with GC from TCGA (excluding patients with OS = 0) into two subgroups of high and low PFRGPS. Kaplan-Meier curve was used to analyze survival status between the high- and low-PFRGPS subgroups. Using the R packages “survival,” “survminer,” and “timeROC,” the receiver operating characteristic (ROC) curves for 3 and 5 years were generated, and the area under the ROC curve (AUC) was calculated to further evaluate the predictive value of PFRGPS. In addition, independent prognosis by the PFRGPS was analyzed *via* univariable and multivariable Cox regression analyses. In three validation cohorts, namely, three independent data sets GSE26901, GSE15459, and GSE84437 from GEO, same process was used to verify the stability of the prognostic model. The PFRGPS established for the TCGA cohort was suitable for the GEO cohort. Before establishing the model, the GEO and TCGA cohorts were processed for batch effect. R packages “survival,” “survminer,” and “timeROC,” were used in the above-mentioned analysis. The R packages used in GEO validation cohorts were consistent with those in the TCGA training cohort.

### Development of a Nomogram Based on the Platelet-Function-Related Genes Prognostic Score and Clinical Factors

Using Cox regression analysis, we constructed a nomogram taking PFRGPS and clinical variables into account, using the R packages “RMS” and “regplot”. In addition, we also generated 3- and 5-year ROC curves. The AUC of the nomogram was calculated to evaluate the prognostic value of the nomogram.

### Gene Set Enrichment Analysis

We performed GSEA on gene expression between the high- and low-PFRGPS subgroups to help understand the related functional differences among different groups. Genome alignment was tested 1,000 times to demonstrate its functional consistency. Phenotypic labels were used to predict adverse events. The file c5.go.v7.4.symbols.gmt was downloaded from the Molecular Signatures Database to run GSEA.

### Analysis of Immune Microenvironment

In order to explore changes in the immune microenvironment in patients with GC, we used the ESTIMATE method to calculate the ImmuneScore and StromalScore of TCGA-cohort samples. ESTIMATEScore is the sum of ImmuneScore and StromalScore.

In order to better clarify the relationship between PFRGPS and immune cell status, immune cells and pathways of each patient were further explored through single-sample GSEA (ssGSEA). We further explore their correlation with PFRGPS and five PPFRGs. In addition, we also used the CIBERSORT algorithm to obtain a relative proportion of 22 kinds of immune cells in each patient so as to quantitatively analyze immune cell infiltration. For the above-mentioned analysis, we used R packages. We used the R packages “limma,” “GSVA,” “GSEABase,” “e1071,” “parallel,” and “preprocessCore” for the above analysis.

### Immunotherapy Analysis

In order to evaluate the response of patients with GC to immunotherapy, we analyzed somatic mutation data using TCGA datasets. The R packages “limma,” “survival,” and “survminer” were used to analyze differences in tumor mutational burden (TMB) between the high- and low-PFRGPS subgroups. Then, TMB was combined with the corresponding survival information to evaluate the relationship between TMB and prognosis. We downloaded the tumor immune dysfunction and exclusion (TIDE), microsatellite instability (MSI), immune dysfunction, immune exclusion, and cancer-associated fibroblast (CAF) scores of patients with GC from the TIDE website (http://tide.dfci.harvard.edu/). Next, MSI status and the immunophenoscore (IPS) of patients with GC were downloaded from The Cancer Immunome Atlas (TCIA; https://tcia.at/home) database. We comprehensively analyzed the effect of immunotherapy on patients with GC and its correlation with PFRGPS using the above-mentioned measures.

### Expression Analysis of PPFRGs

To verify differential expression of PPFRGs between GC and normal tissues, we used the Gene Expression Profiling Interactive Analysis (GEPIA, http://gepia.cancer-pku.cn/) database and the GSE13911 dataset. The GSE13911 dataset was downloaded from the GEO database.

### Statistical Analysis

All statistical analyses in this study were performed using the R software (version 4.1.2). Wilcoxon tests were used to compare differences between two groups. Kaplan-Meier survival analysis was used for comparing OS among different subgroups. ROC curve and AUC were used to evaluate the accuracy of the predictions of the model. Cox regression analysis was used to test independent prognostic characteristics of PFRGPS. Spearman correlation tests were used for correlation analysis. All statistical values with *p* < 0.05 (two-tailed) were considered to be statistically significant.

## Results

### Identification and Functional Enrichment Analysis of DEPFRGs

**FIGURE 2:**
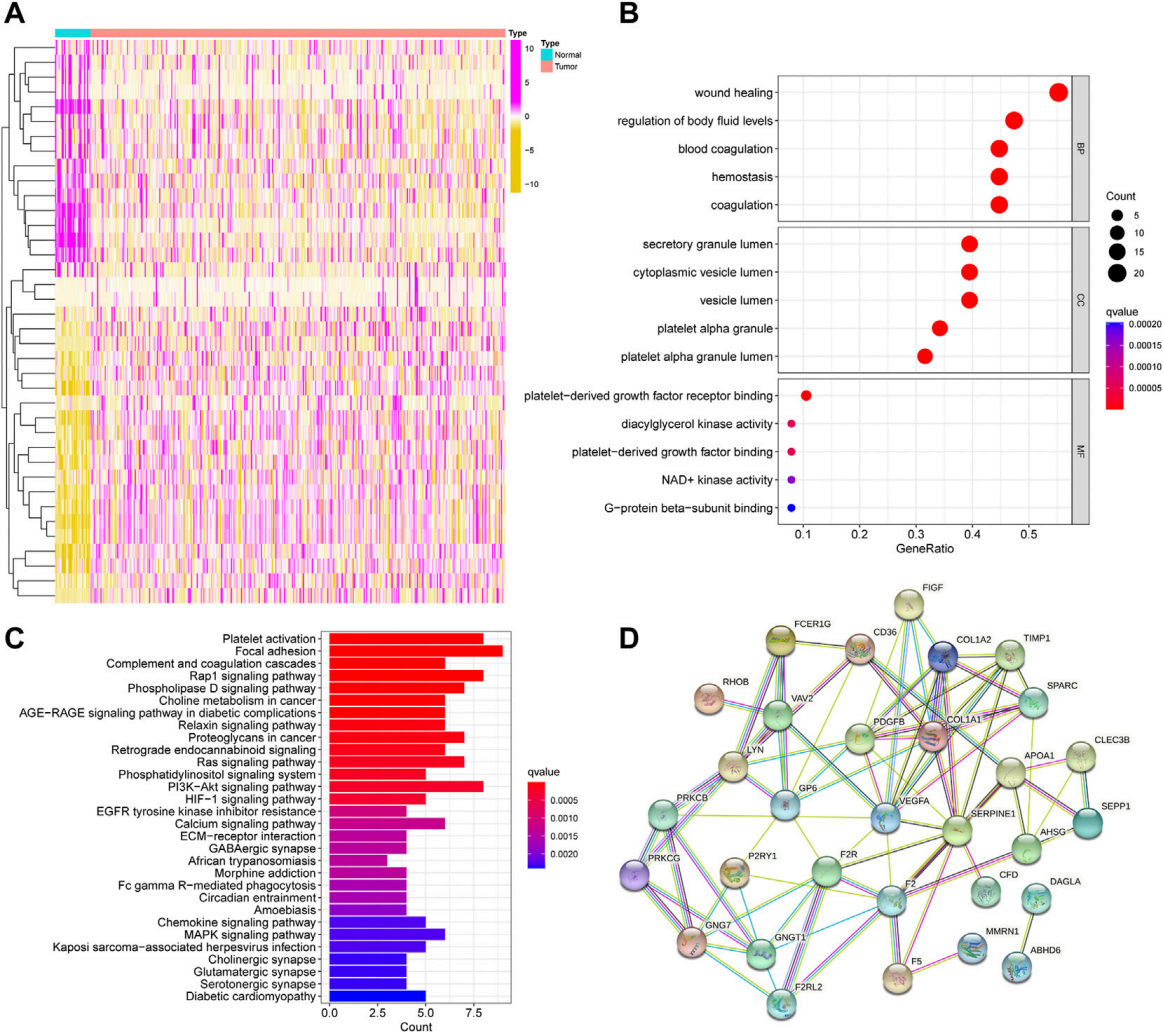
Identification and functional enrichment analysis of DEPFRGs in TCGA cohort. **(A)** The heatmap of 38 DEPFRGs in GC and normal tissues. **(B)** The GO enrichment analysis. **(C)** The KEGG pathway analysis. **(D)** The PPI network was constructed through 38 DEPFRGs. The interaction score was set to 0.4. DEPFRGs, differentially expressed platelet function-related genes; GC, Gastric cancer; GO, Gene Ontology; KEGG, Kyoto Encyclopedia of Genes and Genomes; PPI, protein-protein interaction. *p < 0.05, **p < 0.01, ***p < 0.001.

We found 38 DEPFRGs (22 upregulated and 16 downregulated) in GC and adjacent non-tumor tissues in TCGA cohort (Figure 2A; Supplementary Figures S1A,B). In order to explore the function of DEPFRGs in GC, we first performed GO and KEGG enrichment analyses on DEPFRGs. The GO analysis showed that DEPFRGs were enriched in platelet activation and aggregation, including “wound healing,” “regulation of body fluid levels,” and “blood coagulation” (Figure 2B). Most abundant pathways in the KEGG analysis were related to “platelet activation,” “focal adhesion,” “complement and coagulation cascades,” and “rap1 signaling pathway” (Figure 2C). These findings are related to platelet activation, aggregation, and tumor progression. In addition, we constructed a PPI network with 31 nodes and 81 edges, showing complex interactions among DEPFRGs (Figure 2D).

### Development and Verification of the Platelet-Function-Related Genes Prognostic Score System

**FIGURE 3:**
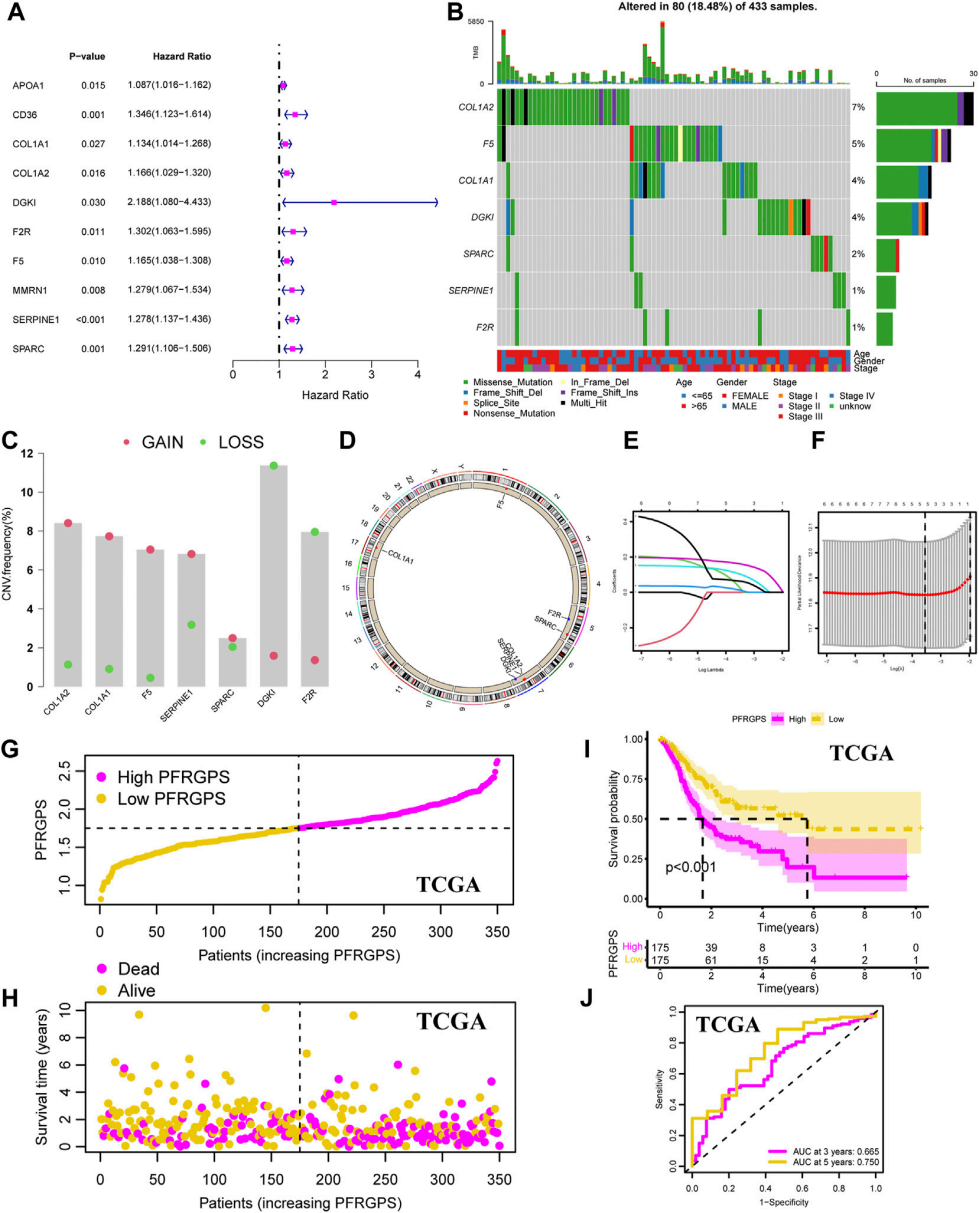
Development of the PFRGPS System. **(A)** The forest plot of 10 PPFGs markers was obtained by univariable Cox analysis. **(B)** The waterfall plot of seven PPFGs mutations. **(C)** Gain (red) or loss (green) CNVs of GC patients. **(D)** The location and CNVs of seven PPFGs. **(E)** LASSO coefficient profiles of seven PPFGs. **(F)** The tuning parameters were cross-validated in the LASSO model. **(G)** Distribution of PFRGPS in TCGA cohort. **(H)** Survival status in the high-PFRGPS and low-PFRGPS subgroups of the TCGA cohort. **(I)** Kaplan-Meier survival analysis in TCGA cohort. **(J)** The ROC curve analysis according to the 3- and 5-year survival of the AUC value in the TCGA cohort. PFRGPS, platelet function-related genes prognostic score; PPFGs, prognosis-related platelet function-related genes; CNVs, copy number variations; LASSO, Least Absolute Shrinkage and Selection Operator; ROC, receiver operating characteristic; AUC, area under the curve.

In order to avoid differences between the gene symbols of GEO verification cohorts and TCGA cohorts, we first considered the intersection of gene symbols of two cohorts. We first performed a univariable Cox regression analysis on the GC group in TCGA cohort and identified 10 PPFRGs (*APOA1, CD36, COL1A1, COL1A2, DGKI, F2R, F5, MMRN1, SERPINE1*, and *SPARC*) that were significantly associated with the OS in patients with GC; high expression of these genes was positively correlated with a poor prognosis (Figure 3A; Supplementary Figure S1C). Compared to that in the adjacent normal tissues, we observed low expression of *ApoA1*, *CD36*, and *MMRN1* in the GC tissues from TCGA dataset. Since this observation was contrary to the results obtained with univariable analysis, we removed these three genes in order to ensure the accuracy of PFRGPS. Finally, seven PFRGs (*COL1A1*, *COL1A2*, *DGKI*, *F2R*, *F5*, *SERPINE1*, and *SPARC*) were selected. In order to confirm the accuracy of these PFRGs, we used the GEPIA database and GSE13911 for verification (Supplementary Figures S2A,B).

In addition, somatic mutation status in GC tissues was analyzed. The results showed that for these seven PFRGs, 80 of 433 GC samples had gene mutations (18.48%), of which missense mutations accounted for largest proportion (Figure 3B). Next, copy number variations (CNVs) in the seven PFRGs were analyzed, and the position of each gene was visualized. Among them, the amplification frequency of CNVs in *COL1A1, COL1A2, F5, SERPINE1*, and *SPARC* was the highest. In contrast, the CNV deletion frequency in *DGKI* and *F2R* was significantly higher than that in the other PFRGs (Figures 3C,D). Next, we performed Lasso regression analysis on the seven selected genes to select the best OS with a non-zero coefficient (Figure 3E). A 10-fold cross-validation was carried out (Figure 3F). According to the minimum standard, we finally selected five PFRGs (*DGKI*, *F2R*, *F5*, *SERPINE1*, and *SPARC*) as genes with independent prognostic significance for PFRGPS system construction. Construction method of PFRGPS: score = 0.034 × expression quantity of *DGKI* + 0.014 × expression quantity of *F2R* + 0.106 × expression quantity of *F5* + 0.1623 × expression quantity of *SERPINE1* + 0.064 × expression quantity of *SPARC*. Considering median PFRGPS of the TCGA cohort as a critical value, PFRGPS was divided into two subgroups: a high-PFRGPS subgroup (*n* = 175) and a low-PFRGPS subgroup (*n* = 175; Figure 3G). In TCGA cohort, patient survival began to decline as the PFRGPS increased (Figure 3H). Kaplan-Meier analysis showed that there was a significant difference in survival between the two subgroups (*p* < 0.001), and the OS of patients with GC in the high-PFRGPS subgroup was significantly lower than that in the low-PFRGPS subgroup (Figure 3I). To further explore the effectiveness of PFRGPS in predicting GC survival, we plotted time-dependent ROC curves for patients with GC with survival periods of 3 and 5 years with AUCs of 0.665 and 0.750, respectively (Figure 3J). In addition, we stratified the patients according to clinicopathological features and found that the PFRGPS was applicable to patients of different ages, genders, and stages (Supplementary Figure S3). These results show that the prediction by PFRGPS is highly specific and sensitive.

### Three Independent GEO Datasets Validate Platelet-Function-Related Genes Prognostic Score

**FIGURE 4:**
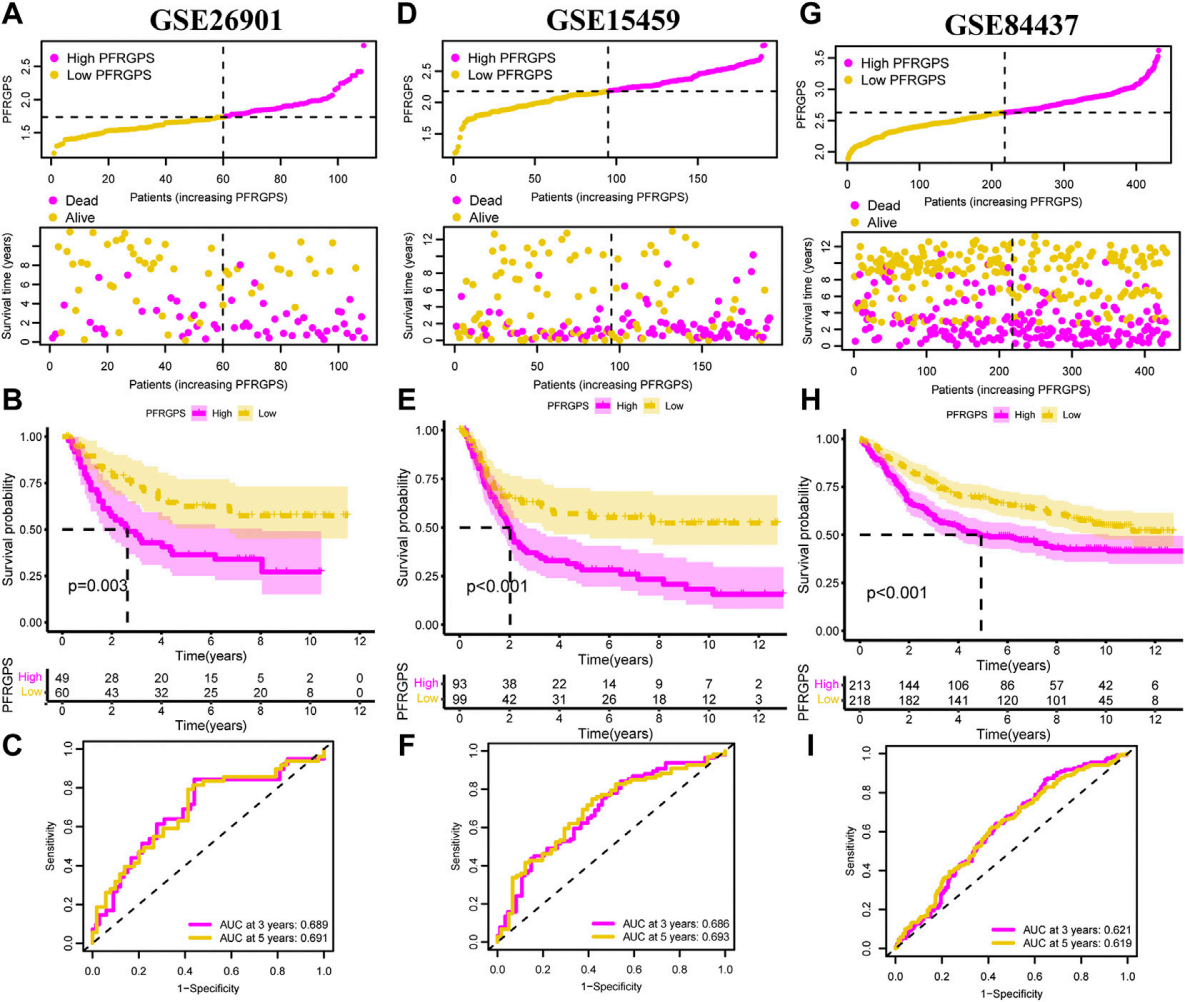
PFRGPS was validated using three independent GEO datasets. In **(A–C)** GSE26901, **(D–F)** GAE15459, and **(G–I)** GSE84437, distribution of PFRGPS and survival status analysis, Kaplan-Meier survival analysis, ROC curve analysis were performed in the high- and low-PFRGPS subgroups. PFRGPS, platelet function-related genes prognostic score; ROC, receiver operating characteristic.

In order to validate the accuracy of PFRGPS in predicting GC, we used the GSE26901 (*n* = 109), GSE15459 (*n* = 191), and GSE84437 (*n* = 431) datasets as external validation cohorts (excluding patients with 0 days follow-up). In these three validation cohorts, patients with GC were divided into high-PFRGPS and low-PFRGPS subgroups according to the median PFRGPS generated in TCGA training cohort (Figures 4A–I). In three validation sets, PFRGPS distribution, survival state, and survival time were consistent with the PFRGPS distribution in TCGA training cohort, suggesting the accuracy of PFRGPS as a prognostic index for GC.

### Independent Prognostic Analysis

**FIGURE 5:**
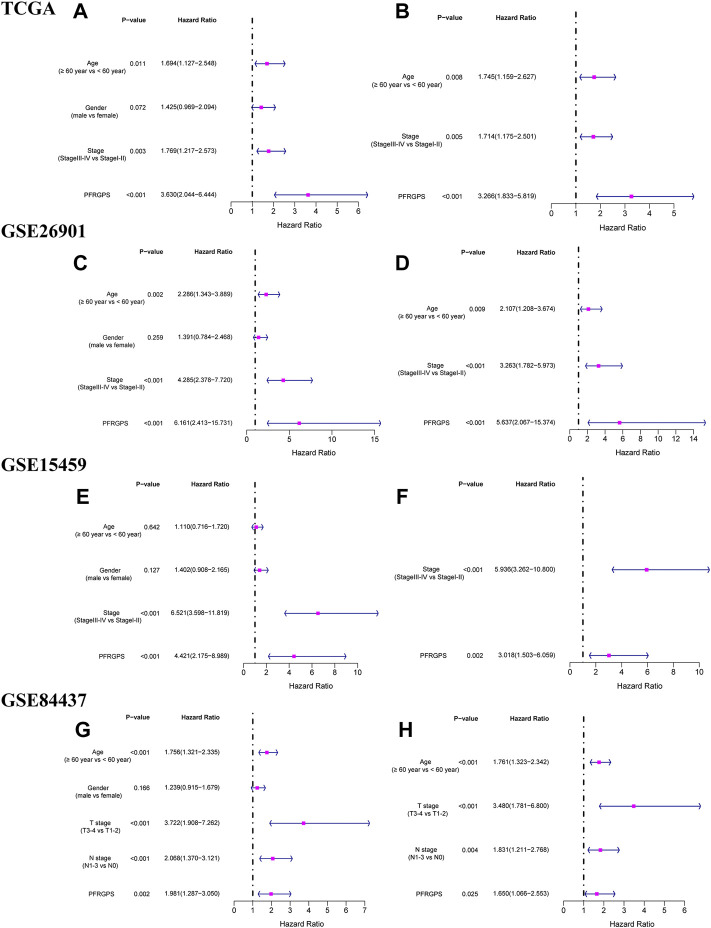
Independent prognostic analyses of prognostic models were performed. The forestplot of univariable Cox regression analysis of PFRGPS and clinical characteristics in **(A)** TCGA, **(C)** GSE62901, **(E)** GSE15459, **(G)** GSE84437. The forestplot of multivariable Cox regression analysis of PFRGPS and clinical characteristics in **(B)** TCGA, **(D)** GSE62901, **(F)** GSE15459, **(H)** GSE84437. PFRGPS, platelet function-related genes prognostic score.

We performed univariable and multivariable Cox regression analysis to evaluate whether PFRGPS is an independent prognostic factor. In TCGA cohort, univariable and multivariable regression analyses of PFRGPS returned hazard ratios (HRs) of 3.203 and 3.266, respectively (*p* < 0.001) (Figures 5A,B). Three GEO cohorts were used for verification, and consistent results were obtained (since no stage was provided in the clinical information of the GSE84437 cohort, we used the T and N stage in clinical information instead) (Figures 5C–H). These analyses show that PFRGPS has an excellent stability and can be used as an independent prognostic factor for patients with GC.

### Nomogram Model and GSEA Analysis

**FIGURE 6:**
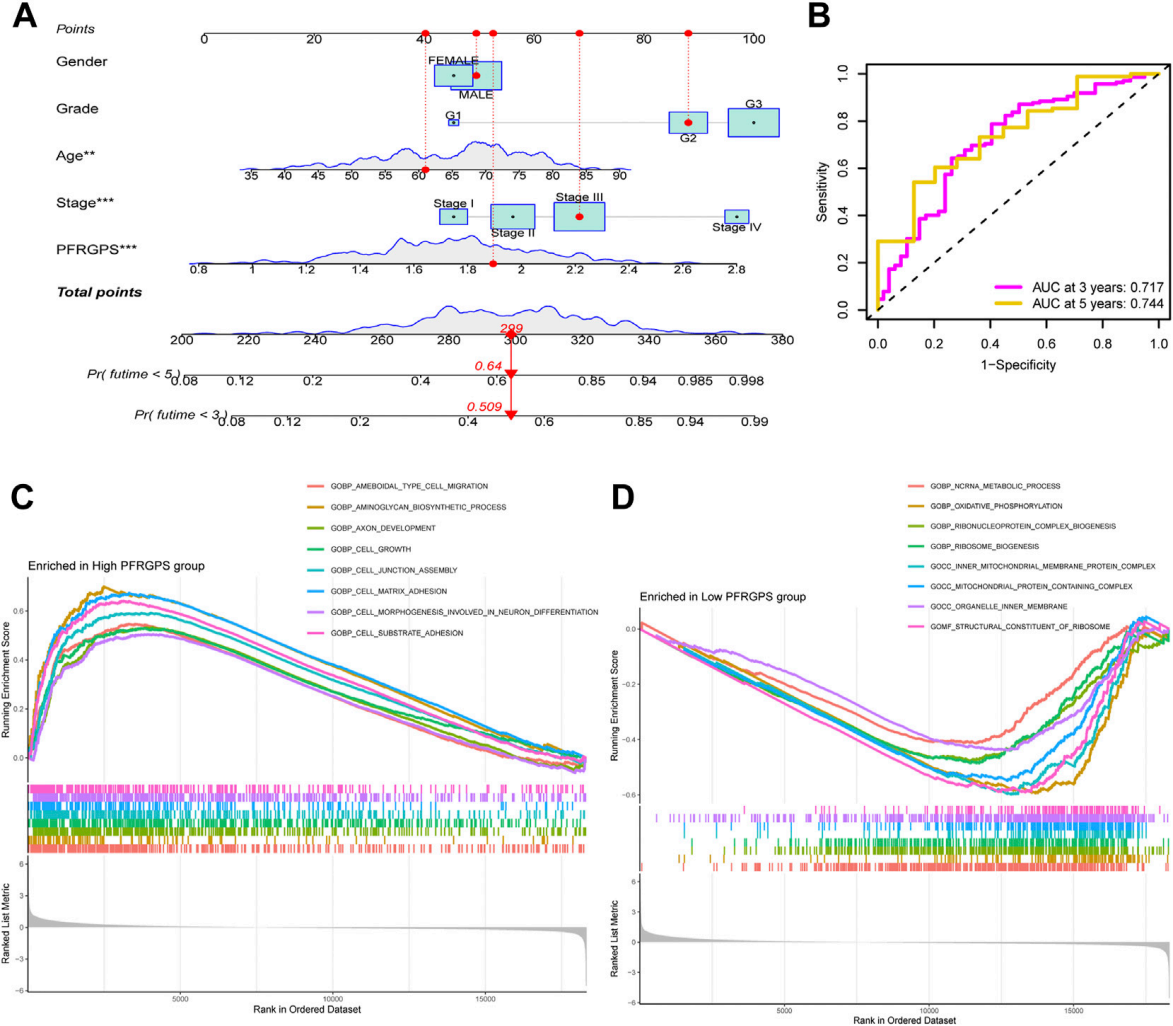
Nomograph Model and GSEA Analysis. **(A)** Nomogram of PFRGPS and clinical factors predicting survival probability of GC patients. **(B)** The ROC curve verifies the predictive ability of the nomogram. **(C)** GSEA enrichment analysis in the high-PFRGPS subgroup. **(D)** GSEA enrichment analysis in the low-PFRGPS subgroup. PFRGPS, platelet function-related genes prognostic score. GSEA, Gene Set Enrichment Analysis; PFRGPS, platelet function-related genes prognostic score; GC, Gastric cancer; ROC, receiver operating characteristic. *p < 0.05, **p < 0.01, ***p < 0.001.

In order to further individualize the prognosis of patients with GC, we established a nomogram model using TCGA cohort and predicted the 3- and 5-year OS (Figure 6A). The ROC curves showed an excellent model sensitivity, with AUC of 0.717 and 0.744 for the 3- and 5-year OS, respectively) (Figure 6B). We further studied different characteristics of biological function activation between the high- and low-PFRGPS subgroups using GSEA. We found that the biological processes enriched in the high-PFRGPS subgroup were “cell growth,” “cell substrate adhesion,” and “cell matrix adhesion” (Figure 6C). The biological processes enriched in the low-PFRGPS subgroup were “ncRNA metabolig process,” and “oxidative phosphorylation,” the enriched cellular components were “inner mitochondrial membrane protein complex,” and “mitochondrial protein containing complex,” and the enriched molecular function was “structural constituent of ribosome” (Figure 6D). These results showed that the high-PFRGPS subgroup was enriched in pathways related to tumorigenesis and progression, further suggesting that PFRGPS can accurately identify tumor progression. Poor prognosis of the high-PFRGPS subgroup was extrapolated from mechanism.

### Tumor Microenvironment Analysis

**FIGURE 7:**
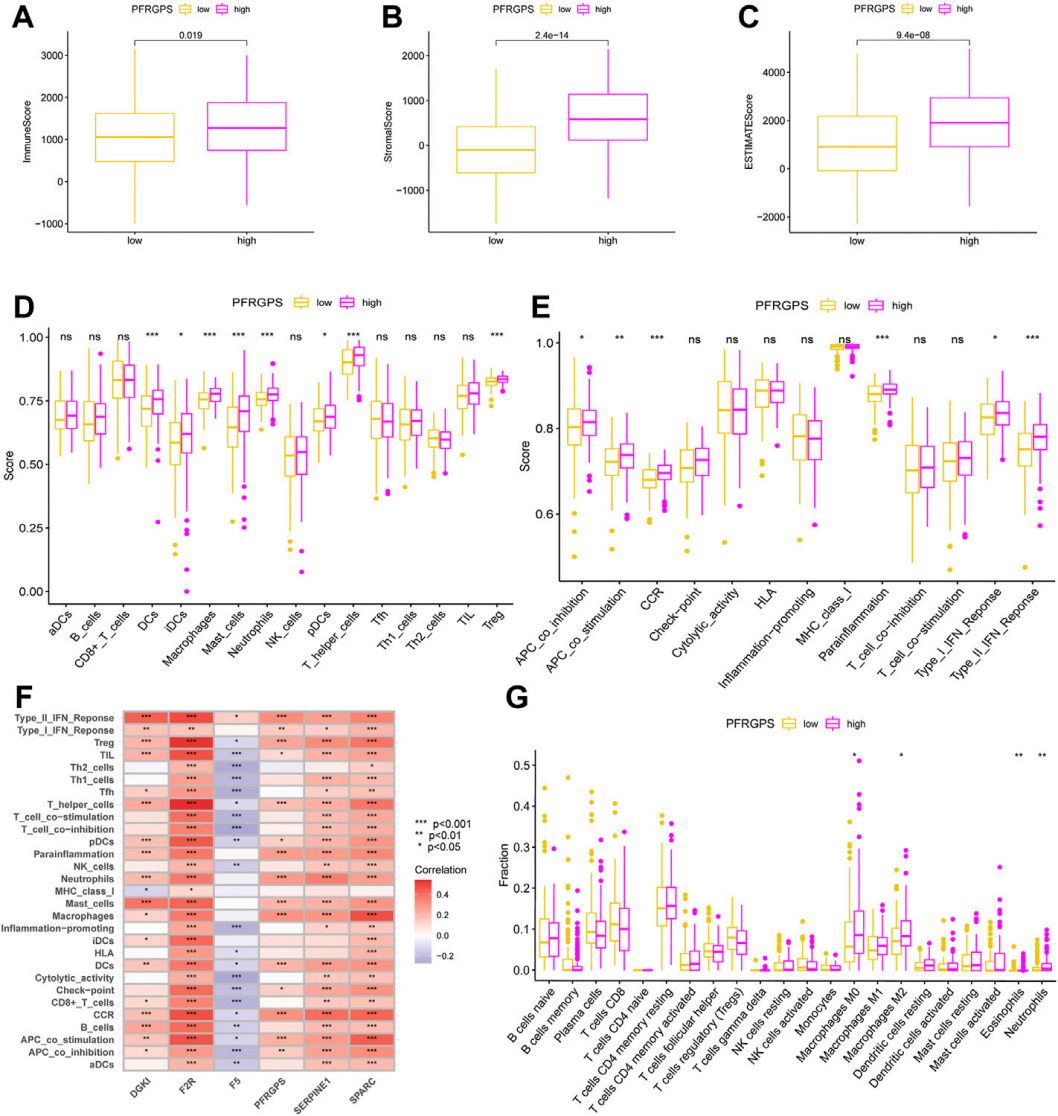
Analysis of tumor microenvironment in TCGA cohort. **(A)** The boxplot of ImmuneScore differences in the low-PFRGPS and high-PFRGPS subgroups. **(B)** The boxplot of StromalScore differences in the low-PFRGPS and high-PFRGPS subgroups. **(C)** The boxplot of ESTIMATEScore differences in the low-PFRGPS and high-PFRGPS subgroups. **(D)** The boxplot of 16 immune cell differences in the low-PFRGPS and high-PFRGPS subgroups. **(E)** The boxplot of 13 immune signaling pathway differences in the low-PFRGPS and high-PFRGPS subgroups. **(F)** Correlation analysis of PFRGPS and five PPFGs with immune cells and signaling pathways. **(G)** The boxplot of 22 immune cell infiltration differences between high- and low-PFRGPS subgroups. PFRGPS, platelet function-related genes prognostic score; PPFGs, prognosis-related platelet.

The TME and the degree of infiltration of immune and stromal cells in tumors contribute significantly to prognosis and have been proposed to be valuable for the diagnosis and prognostic evaluation of tumors. ImmuneScore is a standard test used to quantify the density of T cells and cytotoxic T cells in TMEs; it is of great value in evaluating cancer prognosis. We used the data from TCGA cohort and the ESTIMATE method. The ImmuneScore was distributed between −983.38 and 3,143.92, the StromalScore ranged from −1,730.73 to 2,151.35, and the ESTIMATEScore ranged from −2,266.61 to 4,969.30. The high-PFRGPS subgroup showed a relatively high ImmuneScore and StromalScore when compared to low-PFRGPS subgroup (Figures 7A–C). It is suggested that there are significant differences in TME between the high- and low-PFRGPS subgroups, and there is more immune cell infiltration in the high-PFRGPS subgroups. Next, we observed differences in survival among patients with different ImmuneScores, StromalScores, and ESTIMATEScores. OS decreased significantly in the high-StromalScore group and the high-ESTIMATEScore group (Supplementary Figure S4). In addition, we supplemented PFRGPS for joint analysis. We found that patients with low ImmuneScore, StromalScore, or ESTIMATEScore and who were in the low-PFRGPS subgroup had the highest survival rate, while patients with high ImmuneScore, StromalScore, or ESTIMATEScore and who were in the high-PFRGPS subgroup had the lowest survival rate (Supplementary Figure S4). In order to explore the relationship between PFRGPS and TME in the high- and low-PFRGPS subgroups, we further analyzed immune cells and pathways using ssGSEA. We found that DCs, iDCs, macrophages, mast cells, neutrophils, pDCs, T helper cells, and Treg cells were enriched in the high-PFRGPS subgroup (Figure 7D). The high-PFRGPS subgroup was also enriched in APC co inhibition, APC co-stimulation, CCR, parainflammation, type I and II IFN response pathways (Figure 7E). In addition, we also analyzed the relationship between PFRGPS and five PPFGs and immune cells and pathways. The *DGKI* gene is closely related to mast cells and the type-II interferon response. *F2R*, *SERPINE1*, and *SPARC* are positively related to immune cells and pathways, while *F5* is negatively related to most immune cells and pathways. PFRGPS is closely related to immune cells such as macrophages and mast cells (Figure 7F).

We thus found that more infiltrated cells in the high-PFRGPS subgroup were related to tumor progression and immune escape. We used the CIBERPORT algorithm to confirm that relatively more M0 macrophages, M2 macrophages, eosinophils, and neutrophils related to immune escape were enriched in the high-PFRGP subgroup (Figure 7G).

### Immunotherapy Response Prediction in Multiple Ways

**FIGURE 8:**
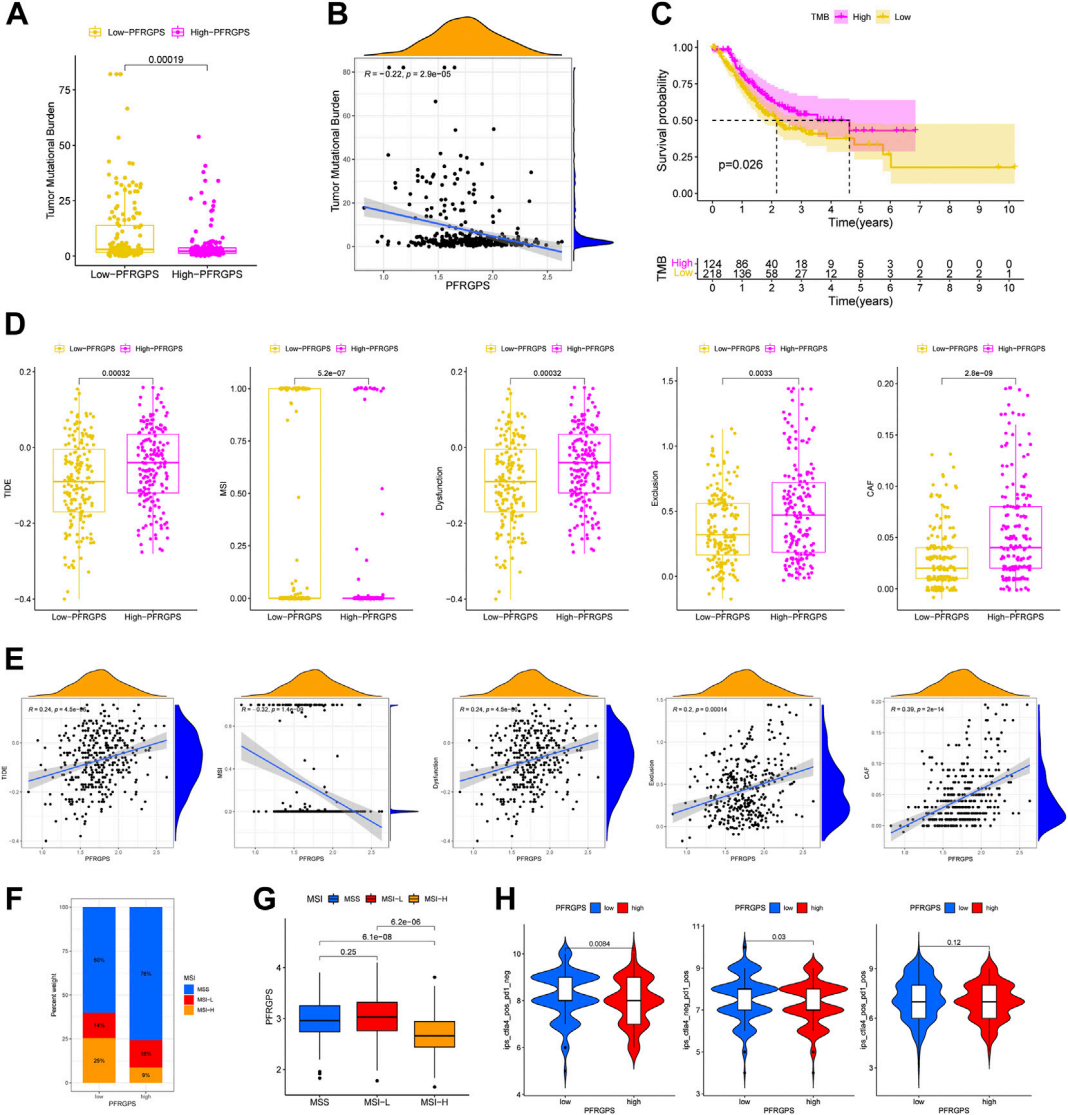
Prediction of immunotherapy response in TCGA cohort. **(A)** The boxplot of TMB differences between low-PFRGPS and high-PFRGPS subgroups. **(B)** Correlation analysis between TMB and PFRGPS. **(C)** Difference in survival time between high- and low-TMB groups. **(D)** The boxplots of differences between TIDE, MSI, Immune Dysfunction, Immune Exclusion, and CAF scores in low- and high-PFRGPS subgroups. **(E)** Correlation analysis of TIDE, MSI, Immune Dysfunction, Immune Exclusion, and CAF scores with PFRGPS. **(F)** Distribution of patients with different MSI statuses in high- and low-PFRGPS subgroups. **(G)** The boxplot of PFRGPS differences between groups with different MSI. **(H)** The violin plots of IPS differences between low-PFRGPS and high-PFRGPS subgroups. TMB, Tumor mutation burden; PFRGPS, platelet function-related genes prognostic score; TIDE, Tumor Immune Dysfunction, and Exclusion; MSI, Microsatellite Instability; CAF, cancer-associated fibroblasts; IPS, immunophenoscore function-related genes. *p < 0.05, **p < 0.01, ***p < 0.001, ns, not significant.

We analyzed differences in somatic mutation distribution between the high- and low-PFRGPS subgroups in TCGA training cohort to explore the relationship between PFRGPS and TMB. We found that TMB was significantly lower in the high-PFRGPS subgroup than in the low-PFRGPS subgroup (Figure 8A). Correlation analysis showed that TMB was negatively correlated with PFRGPS (R = −0.22, *p* < 0.001) (Figure 8B). When compared to that of the high-TMB group, the OS of the low-TMB group was significantly low (Figure 8C). Therefore, our results show that the PFRGPS is consistent with TMB in evaluating the prognosis of patients with GC, which further demonstrates that PFRGPS has an accurate prediction performance.

We used the TIDE score to evaluate the efficacy of immunotherapy. We found that the high-PFRGPS subgroup had relatively high TIDE, immune dysfunction, immune exclusion, and CAF scores and relatively a low MSI score, which suggested the presence of a rich immune escape microenvironment in the high-PFRGPS subgroup (Figure 8D). Next, we found that the TIDE, immune dysfunction, immune exclusion, and CAF scores were positively correlated with PFRGPS, while the MSI score was negatively correlated with NRGPS (Figure 8E). We also found that the high-PFRGPS subgroup had more patients with microsatellite stable (MSS) status, and the high-MSI (MSI-H) group had a lower PFRGPS than the MSS group and low-MSI (MSI-L) group (Figures 8F,G). Therefore, patients with GC in the high-PFRGPS subgroup benefitted less from immunotherapy than patients in the low-PFRGPS subgroup. Next, we used the IPS data obtained from TCIA to predict most commonly used anti-PD-1 and anti-CTLA-4 in ICI therapy. Furthermore, we analyzed potential ICI treatment responses of patients with GC in the high- and low-PFRGPS subgroups. Results showed that the therapeutic effect of anti-PD-1 or anti-CTLA-4 in the low-PFRGPS subgroup was better than that in the high-PFRGPS subgroup (Figure 8H). From these results, it appears that patients with GC in the low-PFRGPS subgroup may respond better to immunotherapy. Patients in the high-PFRGPS subgroup may have fewer exposed immunosuppressant binding sites, potentially leading to a poor prognosis. In the low-PFRGPS subgroup, anti-PD-1 or anti-CTLA-4 alone may yield better efficacy, which also shows that PFRGPS can reliably predict the effectiveness of ICI treatment.

## Discussion

GC is one of most common malignancies of the digestive system. There is increasing evidence that platelets can regulate the TME and promote immune escape and, thus, play an important role in tumor progression and metastasis (Obermann et al., 2021). At present, mechanisms related to PFRG regulation of the immune microenvironment are unclear. Immunotherapy is now widely accepted as a treatment for many types of cancer, including GC (Miliotis and Slack, 2021). However, not all patients can benefit from it. Therefore, there is a need to characterize PFRGs to predict the survival of patients with GC and effective populations for cancer immunotherapy.

We developed a new GC prognostic model, called PFRGPS, using TCGA dataset, and it was independently and externally validated using three GEO datasets. Our results showed that PFRGPS could effectively evaluate the prognosis and clinical status of patients with GC. Our model has higher accuracy than the previous prognostic models that have used PFRGs in tumor lung and breast cancers (Zhou et al., 2021; Xie et al., 2022). Nomograms are widely used for cancer prognosis (Balachandran et al., 2015). Therefore, to further improve the accuracy of prediction, we integrated the PFRGPS, age, gender, tumor grade, and pathological stage to construct and validate our nomogram. Visualization of PFRGPS can help to predict specific survival risk of individual patients, which is of great importance in clinical practice (Zhang et al., 2018).

The GSEA results showed that some classical tumor-associated pathways were significantly enriched in the high-PFRGPS subgroup, indicating that high-PFRGPS is closely related to tumorigenesis and progression. Subsequently, we found that the high-PFRGPS subgroup was enriched with a large number of immunosuppression-related immune cells, revealing a close association with tumor immune escape. We further confirmed the predictive ability of PFRGPS in immunotherapy efficacy by analyzing the TMB, TIDE, microsatellite instability, IPS, PD-1, and CTLA4 models. Our results demonstrate that PFRGPS has satisfactory accuracy, sensitivity, and authenticity.

PFRGPS includes five mRNAs related to platelet function, namely *DGKI*, *F2R*, *F5*, *SERPINE1*, and *SPARC*, all of which are expressed more in GC tissues than in paracancerous ones, and their expression levels are positively correlated with poor prognosis in patients with GC. *DGKI* can expressed in the cytoplasmic matrix of human platelets (Yada et al., 1990); additionally, *DGKI* was recently found to be overexpressed in a variety of cancers, including GC (Huang et al., 2020). Results of basic experimental studies suggest that MAPK signaling may be a key pathway associated with DGKI regulation in GC (Rigg et al., 2019). Coagulation F2R, also known as protease-activated receptor (PAR)-1, is a member of the PAR family, and F2R activation through activation of G proteins can lead to platelet activation, adhesion, and aggregation (Rigg et al., 2019). F2R activation may facilitate platelet activation, tumor cell proliferation, apoptosis, and angiogenesis (Ray and Pal, 2016; Wojtukiewicz et al., 2019; Chang et al., 2020). F2R was found to enhance GC cell invasion, proliferation, and angiogenesis via the nuclear factor kappa B and ERK1/2 signaling pathways in a study of GC (Fujimoto et al., 2010). Coagulation factor V (F5) is a circulating high-molecular-weight (330 kDa) pro-coagulation factor (Cramer and Gale, 2012). F5 was recently found to be capable of being expressed in extravascular tissues, including breast cancer cells and tumor-permeable immune cells (Tinholt et al., 2020). Many studies have reported the association of F5 polymorphisms with the risk of developing various cancers, including colon and gastric cancers (Tinholt et al., 2016). Serine protease inhibitor family E member 1 (SERPINE1) is a major inhibitor of tissue fibrinogen activator and urokinase (Huang et al., 2012) and is associated with the development and progression of a variety of tumors (Saidak et al., 2021). SERPINE1 may regulate VEGF and IL-6 expression through the VEGF signaling pathway and the JAK-STAT3 inflammatory signaling pathway, ultimately affecting GC cell invasion and migration (Chen et al., 2022). As an oncogene, it may promote the proliferation, migration, and invasion of GC tumor cells by mediating the epithelial-mesenchymal transition (Yang et al., 2019). Secreted protein acidic and rich in cysteine (SPARC) is a protein encoded by a single gene in human chromosome 5q31.1 (Termine et al., 1981). SPARC is a matricellular protein that regulates cell adhesion, extracellular matrix production, growth factor activity, and the cell cycle (Sage, 2003). SPARC is a major contributor to tumor progression, drug resistance, and metastasis (Nagaraju et al., 2014). SPARC is markedly upregulated in gastric tissue (Liao et al., 2018). In summary, the use of PFRGPS as a prognostic predictor for patients with GC has a broad research base.

TMEs have been shown to be important in anti-tumor immunity (Murciano-Goroff et al., 2020). The platelets in TMEs have the ability to regulate tumor immune escape (Rachidi et al., 2017). We used three different algorithms in this study to show that the high-PFRGPS subgroup had relatively high ImmuneScores and StromalScores. While the high-ESTIMATEScore PFRGP patients had high ImmuneScore and StromalScore, they had the lowest survival rates. In addition, there was a significant difference in the abundance of immune cell infiltration between the high-PFRGPS and low-PFRGPS subgroups.

Patients in the high-NRGPS subgroup showed a relatively high infiltration of immune cells, among which Treg, M2, and neutrophils are well known immunosuppressive cells, while the role of Tfh in TME is not yet clear. Some studies have found that a high expression of Tfh is positively correlated with the survival of patients with breast, lung, and colorectal cancers (Bindea et al., 2013; Gu-Trantien et al., 2013; Germain et al., 2014). However, in another study based on a mouse model of hepatocellular carcinoma, Tfh cells were found to be negatively associated with survival (Shalapour et al., 2017). We found that Tfh was more infiltrated in the high-PFRGPS subgroup; hence, it is clear that there are interesting complexities associated with Tfh in the context of cancer.

Thus, based on differences in the immune microenvironment between the high- and low-PFRGPS subgroups, it is reasonable to speculate that there may be differences in the effects of immunotherapy between these two subgroups. Both basic research and clinical practice have recently shown that cancer patients who respond to immunotherapy have durable efficacy and longer OS than those who do not respond (Gettinger et al., 2018). Therefore, identifying which patients could benefit from immunotherapy is an important issue.

TMB is the number of genetic mutations within a tumor. TMB is a stable predictor of immunotherapy success, and thus expression of predictive marker is consistent with TMB alterations in order to have high reliability (Chan et al., 2019). Increased TMB is found in a majority of cancer patients who benefit from immunotherapy (Zhao et al., 2019). Consistent with this, we found that the low-PFRGPS subgroup had a higher TMB. The TIDE has a higher accuracy than PD-1 expression levels and TMB in predicting immunotherapy outcomes in cancer patients. A lower TIDE score suggests that a patient may derive greater benefits from immunotherapy; our results are in agreement with this, since we found that the high-PFRGPS group did not benefit much from immunotherapy (Jiang et al., 2018). MSI is a molecular signature of cancer that usually occurs when DNA mismatch repair (dMMR) is disrupted (Boland and Goel, 2010). Evidence is mounting that MSI status affects the survival and treatment of patients with several cancers, including GC (Polom et al., 2018). It is reported that the majority of tumors in Chinese patients with GC (about 95%) are characterized by high MSS (Li et al., 2021). Patients with GC in the MSI-H group had higher survival rates than patients in the MSS or low-MSI groups (Zhang et al., 2022). In patients with colon cancer, it was found that patients in the MSI-H group benefited significantly more from immunotherapy than patients in the MSS or MSI-L groups (Cao et al., 2022). Our study found that patients with GC in the high-PRFGPS subgroup had a higher proportion of MSS and a lower proportion of MSI-H. This may suggest that the high-PRFGPS subgroup had a relatively poor prognosis and poorer immunotherapeutic outcomes. ICI has emerged as a potentially effective cancer treatment (Llovet et al., 2018). Targeting immune checkpoint molecules such as PD-1 and CTLA-4 can reinvigorate anti-tumor immunity (Choi et al., 2019). In order to predict the effect of ICI treatment in patients with GC, we analyzed the relationship between PFRGPS and both PD-1 and CTLA4 using IPS in patients with GC. We found that the IPS scores of anti-CTLA4 or anti-PD-1 were higher in the low-PFRGPS subgroup than in the high-PFRGPS subgroup, which means that immunotherapy may be more effective in the low-PFRGPS subgroup. There was no difference in IPS between the high- and low-PFRGPS subgroups during combined treatment.

Our study has some limitations. Firstly, we only used data from databases and did not perform relevant experimental validation. Secondly, the mechanism of platelet action in the TME of patients with GC is still unclear, with a view to future experimental studies.

Our results showed that, relative to the low-PFRGPS subgroup, the high-PFRGPS subgroup had a pro-cancer immune microenvironment, low TMB, high TIDE, high MSS, low MSI-H characteristics, and relatively poor anti-PD-1 and anti-CTLA-1 therapeutic effects, suggesting that the high-PFRGPS subgroup was associated with immune escape in GC. Therefore, PFRGPS could be used as a new tool to effectively evaluate the prognosis of and immunotherapeutic efficacy in patients with GC.

## Conclusion

Our study defined a novel prognostic signal consisting of five platelet activation, signaling and aggregation-related platelet function-related genes, which was independently and externally validated using three GEO datasets GSE26901, GSE15459, and GSE84437. The signal was independently associated with OS in both the TCGA cohort and the GEO validation cohort, and further demonstrated GC prognosis and immunotherapy efficacy. It can be used as a predictive tool for the selection and outcome of clinical therapies for GC patients.

## Data Availability

The original contributions presented in the study are included in the article/Supplementary Material, further inquiries can be directed to the corresponding author.
