# Supplementary figures and images for: Characterization of Platelet Function-Related Gene Predicting Survival and Immunotherapy Efficacy in Gastric Cancer
Source: Front Genet. 2022 Jun 28;13:938796. doi: 10.3389/fgene.2022.938796 (PMC9274243; doi:10.3389/fgene.2022.938796)

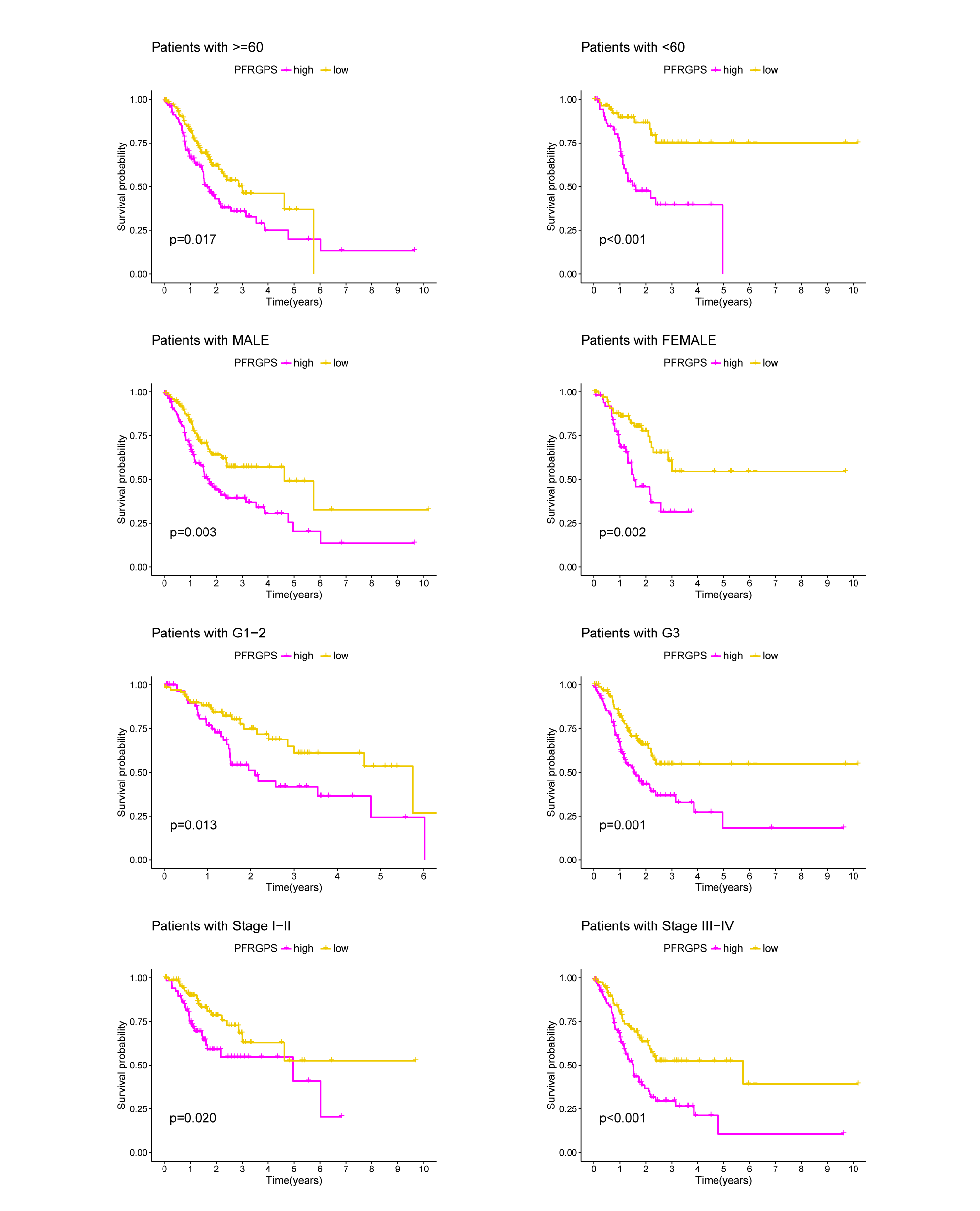

Supplement: Supplementary file 4 [file Image3.TIF]

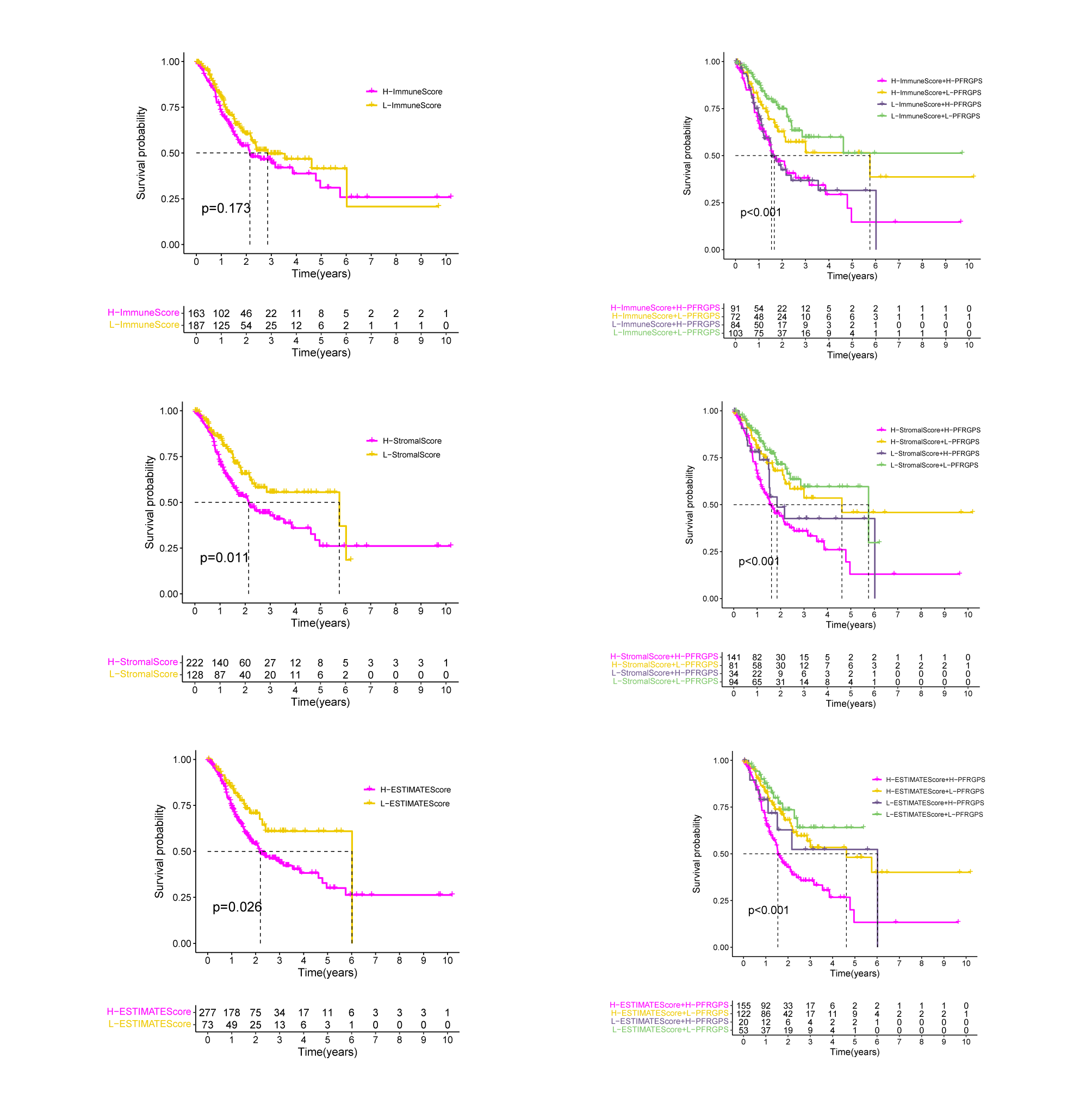

Supplement: Supplementary file 5 [file Image4.TIF]

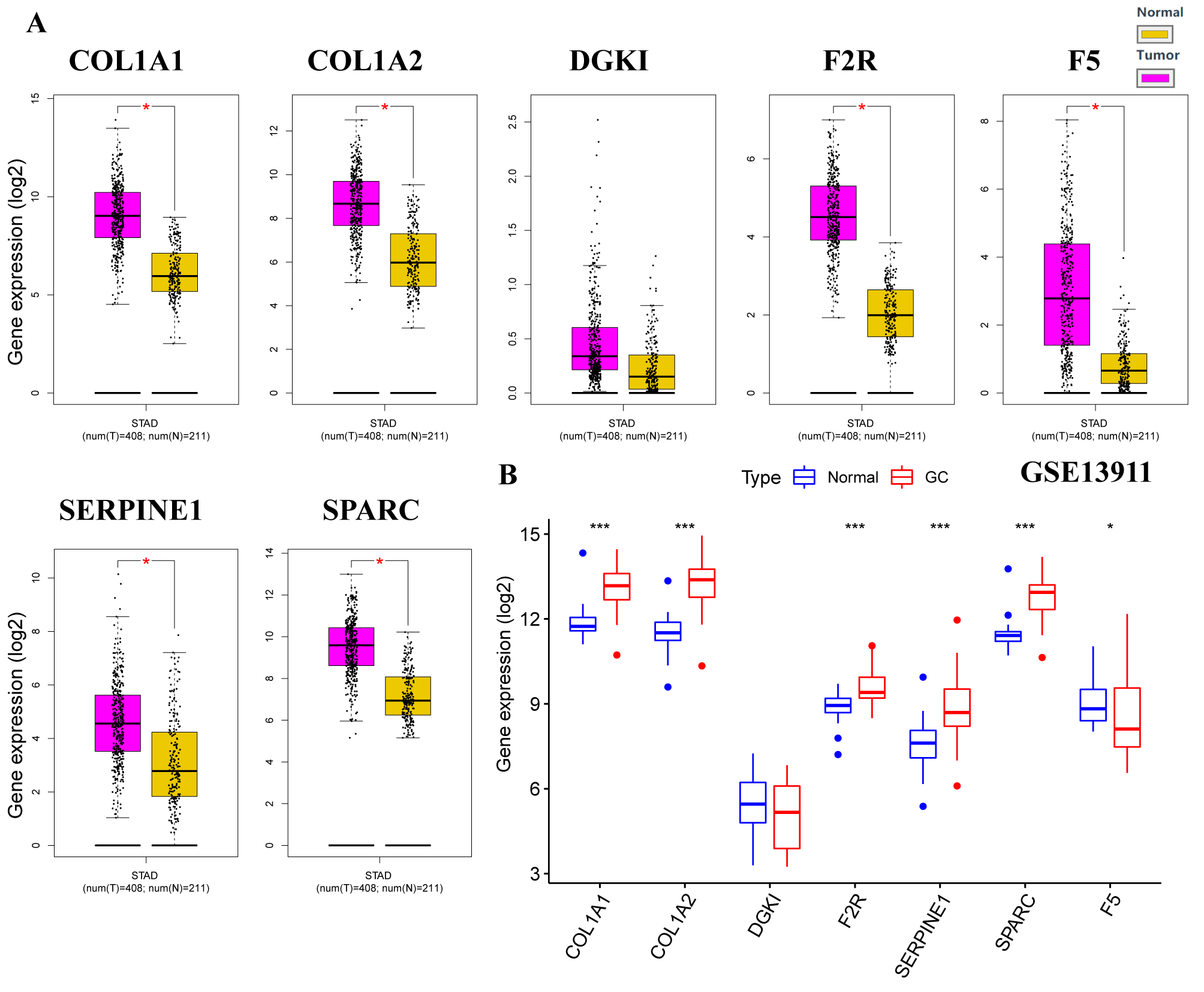

Supplement: Supplementary file 6 [file Image2.TIF]

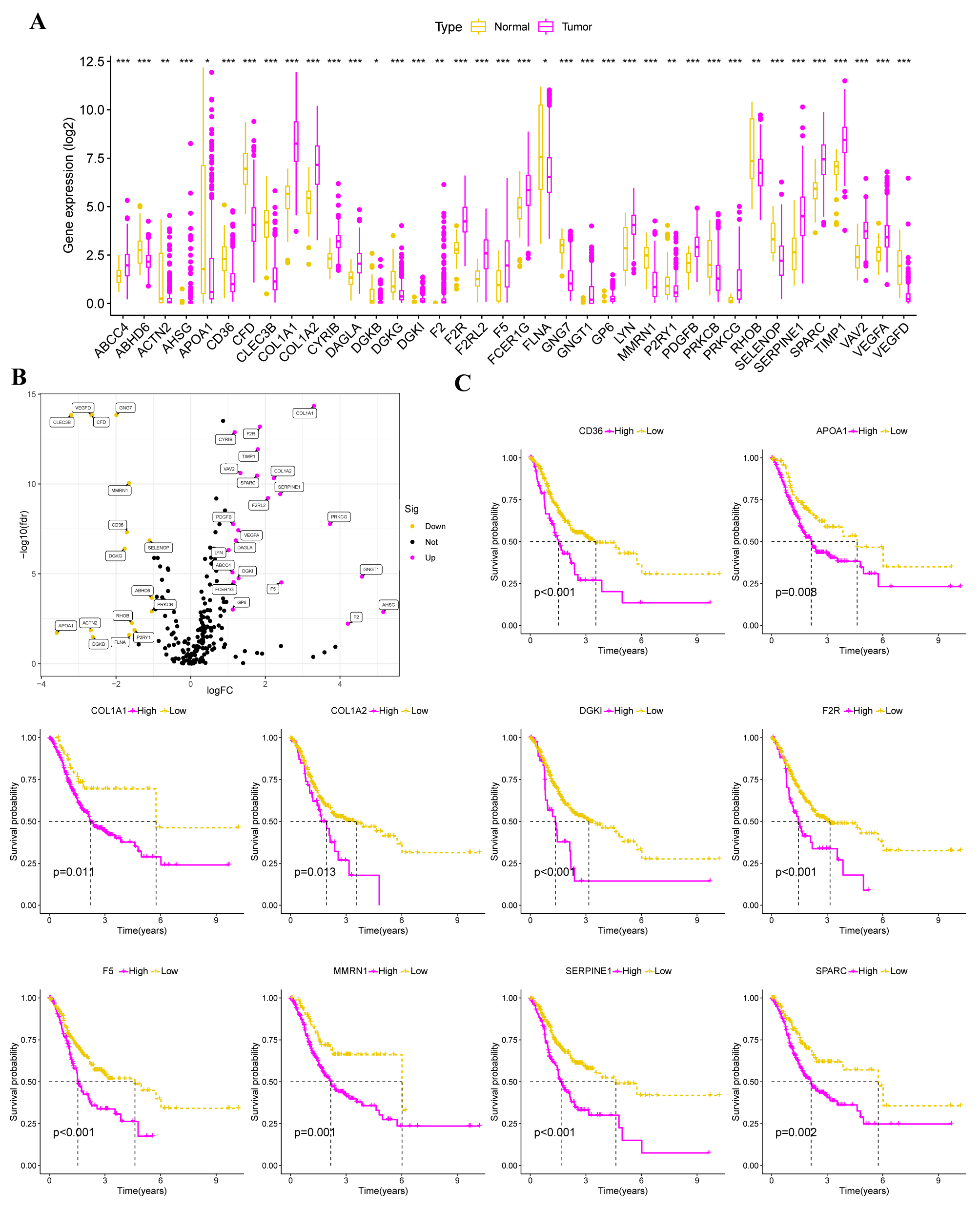

Supplement: Supplementary file 7 [file Image1.TIF]
