# Supplementary material for: Characterization of Platelet Function-Related Gene Predicting Survival and Immunotherapy Efficacy in Gastric Cancer
Source: Front Genet. 2022 Jun 28;13:938796. doi: 10.3389/fgene.2022.938796 (PMC9274243; doi:10.3389/fgene.2022.938796)
Supplement: Supplementary file 14 [file DataSheet1.docx]

Supplementary Material

# Supplementary Figure S1. Analysis of the relationship between the expression of 10 PPFGs and the survival rate of GC patients.


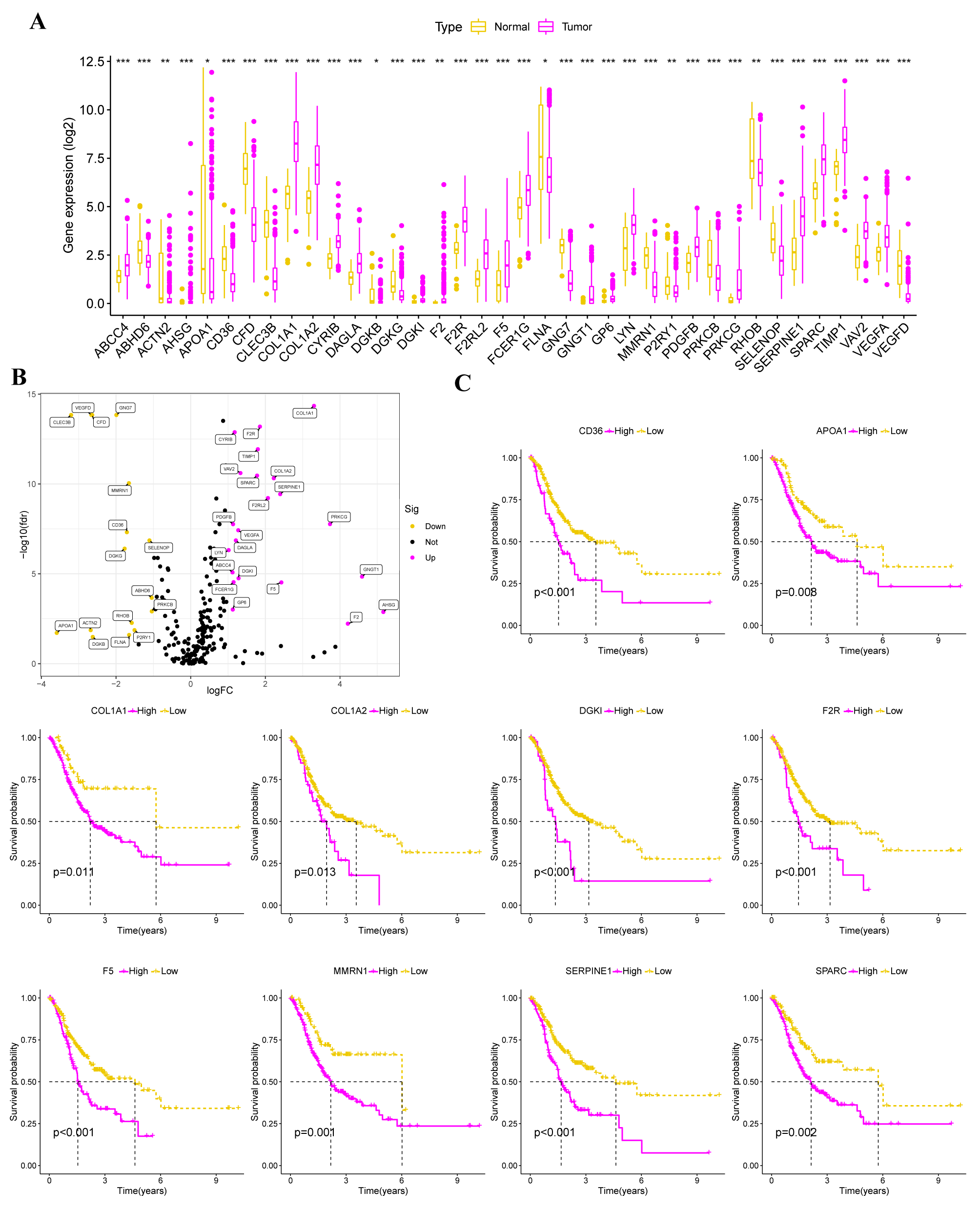


FIGURE S1| Analysis of the relationship between the expression of 10 PPFGs and the survival rate of GC patients.

(A) The boxplot of 38 DEPFRGs in GC and normal tissues.

(B) The volcano plot of 38 DEPFRGs in GC and normal tissues.

(C) Kaplan-Meier survival curve of OS in patients with high and low APOA1, CD36, COL1A1, COL1A2, DGKI, F2R, F5, MMRN1, SERPINE1, and SPARC expression.

DEPFRGs, differentially expressed platelet function-related genes; PPFGs, prognosis-related platelet function-related genes; GC, Gastric cancer.

# Supplementary Figure S2. PPFGs were verified in the GEPIA database and GSE13911 dataset.


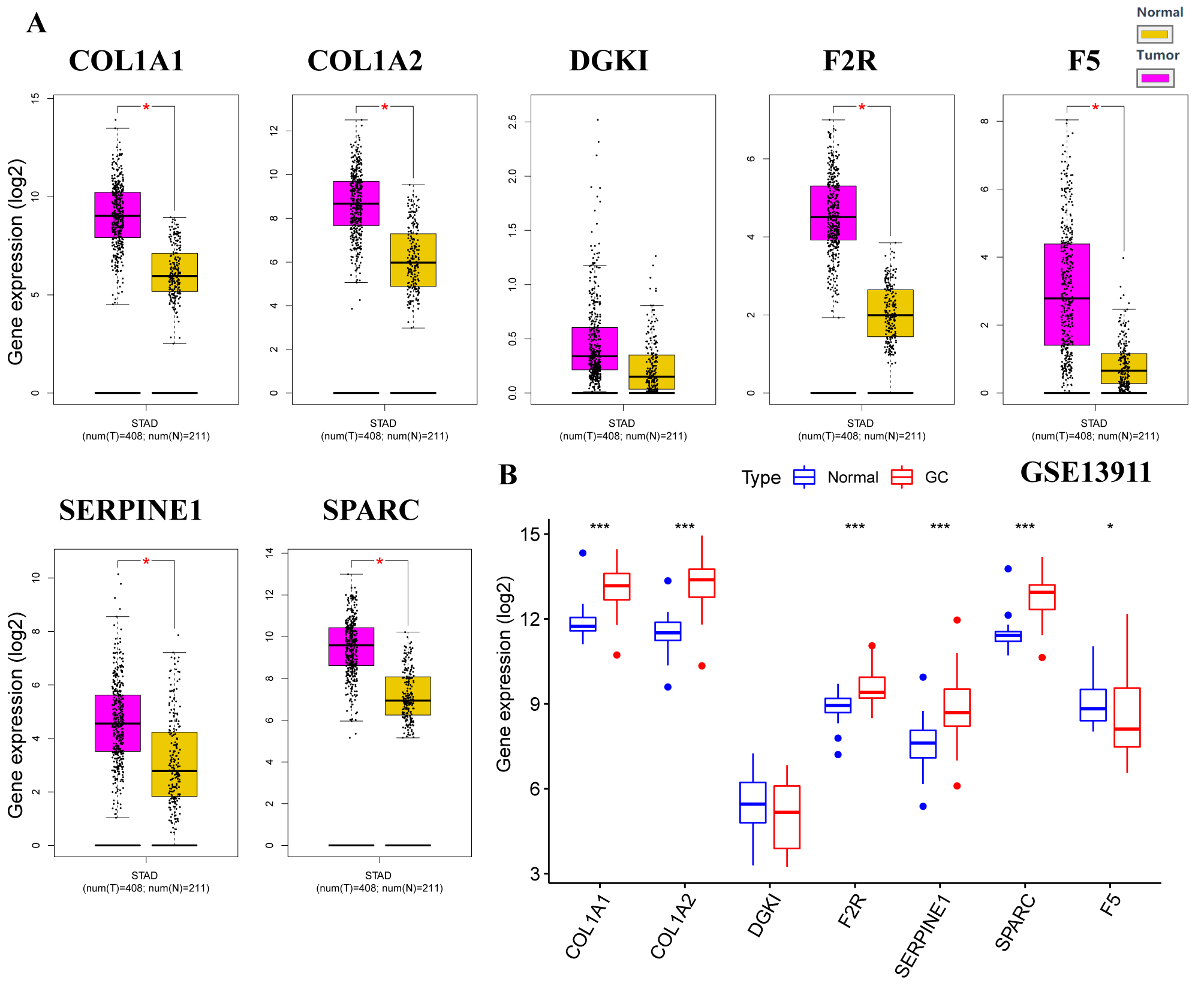


FIGURE S2| PPFGs were verified in the GEPIA database and GSE13911 dataset.

(A) The boxplots of PPFGs expression in normal and GC tissues in the GEPIA database.

(B) The boxplot of PPFGs expression in normal and GC tissues in the GSE13911 dataset.

PPFGs, prognosis-related platelet function-related genes; GC, Gastric cancer. **p* < 0.05, ***p* < 0.01, ****p* < 0.001.

# Supplementary Figure S3. Kaplan-Meier survival curve based on clinical characteristics stratification of GC patients in the TCGA cohort.

#
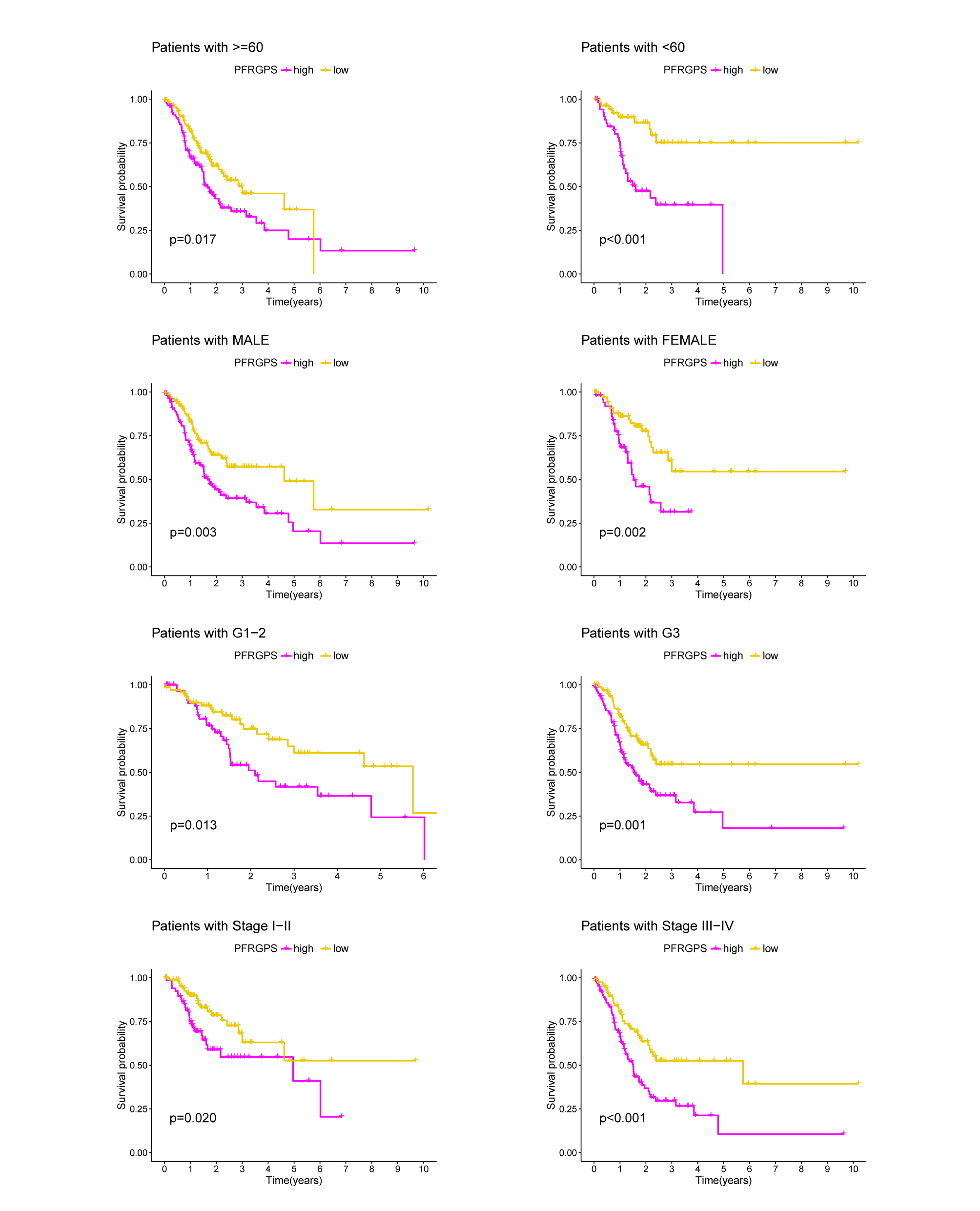


FIGURE S3| Kaplan-Meier survival curve based on clinical characteristics stratification of GC patients in the TCGA cohort.

GC, Gastric cancer.

# Supplementary Figure S4. Survival analysis of ImmuneScore, StromalScore, or ESTIMATEScore combined with PFRGPS.


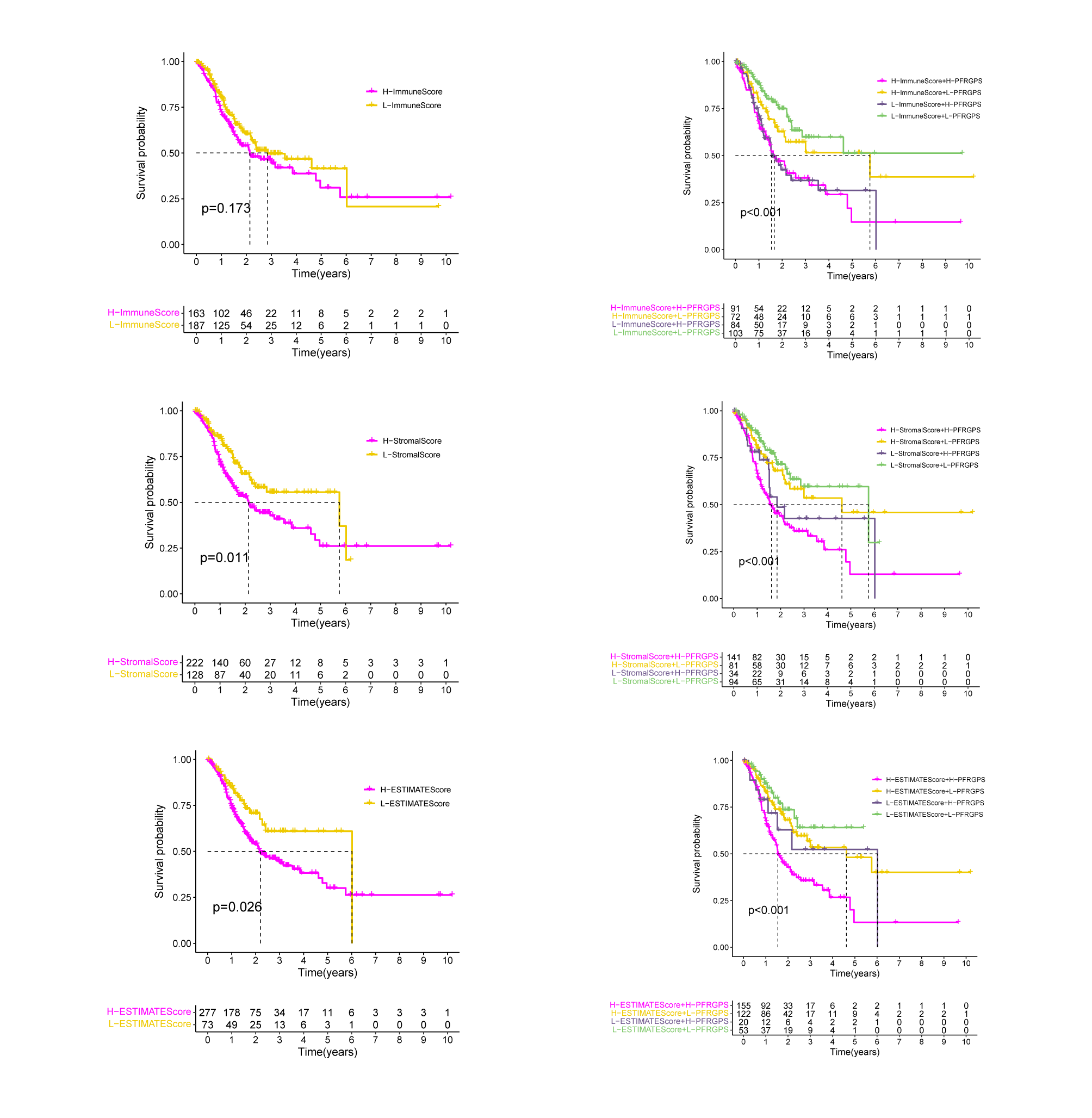


# Supplementary Table S1. Patients’ clinical features of TCGA and GEO cohorts.

| Variables | | TCGA (n = 350) | | GSE26901 (n = 109) | | GSE84437 (n = 431) | | GSE15459 (n = 191) | |
| --- | --- | --- | --- | --- | --- | --- | --- | --- | --- |
|  |  | Number | Percentage (%) | Number | Percentage (%) | Number | Percentage (%) | Number | Percentage (%) |
| Age | ≥ 60 | 238 | 68.00 | 45 | 41.28 | 249 | 57.77 | 132 | 69.11 |
|  | < 60 | 109 | 31.14 | 64 | 58.72 | 182 | 42.23 | 59 | 30.89 |
|  | Unknow | 3 | 0.86 | 0 | 0.00 | 0 | 0.00 | 0 | 0.00 |
| Gender | Male | 226 | 64.57 | 69 | 63.30 | 294 | 68.21 | 124.00 | 64.92 |
|  | Female | 124 | 35.43 | 40 | 36.70 | 137 | 31.79 | 67.00 | 35.08 |
| Survival status | Alive | 207 | 59.14 | 54 | 49.54 | 224 | 51.97 | 96 | 50.26 |
|  | Dead | 143 | 40.86 | 55 | 50.46 | 207 | 48.03 | 95 | 49.74 |
| Grade | G1 | 9 | 2.57 | 0 | 0.00 | 0 | 0.00 | 0 | 0.00 |
|  | G2 | 125 | 35.71 | 0 | 0.00 | 0 | 0.00 | 0 | 0.00 |
|  | G3 | 207 | 59.14 | 0 | 0.00 | 0 | 0.00 | 0 | 0.00 |
|  | Unknow | 9 | 2.57 | 0 | 0.00 | 0 | 0.00 | 0 | 0.00 |
| Clinical stage | Stage I | 46 | 13.14 | 40 | 36.70 | 0 | 0.00 | 31 | 16.23 |
|  | Stage II | 110 | 31.43 | 18 | 16.51 | 0 | 0.00 | 29 | 15.18 |
|  | Stage III | 145 | 41.43 | 36 | 33.03 | 0 | 0.00 | 72 | 37.70 |
|  | Stage IV | 35 | 10.00 | 15 | 13.76 | 0 | 0.00 | 59 | 30.89 |
|  | Unknow | 14 | 4.00 | 0 | 0.00 | 0 | 0.00 | 0 | 0.00 |
| Tstage | T1 | 16 | 4.57 | 0 | 0.00 | 11 | 2.55 | 0 | 0.00 |
|  | T2 | 74 | 21.14 | 0 | 0.00 | 38 | 8.82 | 0 | 0.00 |
|  | T3 | 161 | 46.00 | 0 | 0.00 | 92 | 21.35 | 0 | 0.00 |
|  | T4 | 95 | 27.14 | 0 | 0.00 | 290 | 67.29 | 0 | 0.00 |
|  | Unknow | 4 | 1.14 | 0 | 0.00 | 0 | 0.00 | 0 | 0.00 |
| Mstage | M0 | 312 | 89.14 | 102 | 93.58 | 0 | 0.00 | 0 | 0.00 |
|  | M1 | 23 | 6.57 | 7 | 6.42 | 0 | 0.00 | 0 | 0.00 |
|  | Unknow | 15 | 4.29 | 0 | 0.00 | 0 | 0.00 | 0 | 0.00 |
| Nstage | N0 | 103 | 29.43 | 0 | 0.00 | 80 | 18.56 | 0 | 0.00 |
|  | N1 | 93 | 26.57 | 0 | 0.00 | 187 | 43.39 | 0 | 0.00 |
|  | N2 | 72 | 20.57 | 0 | 0.00 | 132 | 30.63 | 0 | 0.00 |
|  | N3 | 71 | 20.29 | 0 | 0.00 | 32 | 7.42 | 0 | 0.00 |
|  | unknow | 11 | 3.14 | 0 | 0.00 | 0 | 0.00 | 0 | 0.00 |
